# Supplementary material for: Multi-centric evaluation of Biomeme Franklin Mobile qPCR for rapid detection of Mycobacterium ulcerans in clinical specimens
Source: PLoS Negl Trop Dis. 2023 May 25;17(5):e0011373. doi: 10.1371/journal.pntd.0011373 (PMC10246808; doi:10.1371/journal.pntd.0011373)
Supplement: Attachment — Submitted filename: Feedback_DMP_02022023.docx [file pntd.0011373.s003.docx]

**General comment:**

We congratulate the authors on a research topic that is relevant in several ways. It is one of the research priorities for Buruli ulcer control. This study addresses several aspects of control, such as decentralizing molecular biology to improve access to rapid diagnostic confirmation for affected individuals, improving patient management and reducing the cost of care.

**Specific comments:**

- **Ethical considerations**: Taking into account that this study involving human beings is multi-centre, especially multi-country, ethical approval should normally be obtained in each country. The authors only refer to the approval obtained in Ghana. What about Cameroon?
- The first two phases were carried out in reference laboratories. Are the authors not planning a third phase of the study? Since they believe that this innovative diagnostic tool has the potential to be fully used in primary care settings, wouldn't it make sense to test them at this level of care as well?
- The limiting factor, not the least, for the expansion of this technology is the cost per test ($20-25). Innovation obviously has a cost. But from an operational point of view, who will be able to pay for this test when we know that the people affected are generally poor and live in disadvantaged environments? This is a crucial aspect that should be considered by the authors. They only mentioned that an alternative, cheaper extraction kit could be used, without saying more.
