# Supplementary material for: Multi-centric evaluation of Biomeme Franklin Mobile qPCR for rapid detection of Mycobacterium ulcerans in clinical specimens
Source: PLoS Negl Trop Dis. 2023 May 25;17(5):e0011373. doi: 10.1371/journal.pntd.0011373 (PMC10246808; doi:10.1371/journal.pntd.0011373)
Supplement: Attachment — Submitted filename: Coverletter_response.doc [file pntd.0011373.s004.doc]

April 2, 2023

Editors

PLOS Neglected Tropical Diseases

**Authors responses to reviewers’ comments**

Authors appreciate the comments of reviewers for their support to improve our manuscript. We have indicated below our responses (in blue text) to the various comments and description of the changes made to the manuscript below.

Reviewer #1: The present work deals with the presentation of a miniaturized real-time PCR system plus DNA extraction method and its comparison to conventional qPCR method used in the diagnosis of Buruli ulcer (BU). PCR is the method recommended by the World Health Organization (WHO) to confirm cases of BU. In principle this miniaturized PCR would allow decentralized diagnosis enabling early treatment, and the contributing to WHO goals.
The work is relevant, but I believe there are some aspects n the manuscript that would need to be addressed:

1- In the abstract and introduction the authors indicate that current PCR methods are not as able to be implemented in decentralized settings as it would be this miniaturized PCR. However, data on usability (DNA extraction and PCR methods) are not presented. This makes difficult to see the potential advantages of this new PCR system. This is also part of the discussion, but data or work addressing usability is missed.

**Response:** Authors agree with reviewer that data on an extraction method will help decentralized this miniaturized PCR system for *M. ulcerans* detection. But this current manuscript is focus on the usability of this PCR platform for detection of *M. ulcerans* comparison to a standard PCR platform using stored samples. Authors intend to follow this study with an onsite evaluation of this system together with a field-friendly extraction method.

2- Abstract. Indicate sensitivity and specificity against which reference test.

**Response:** The reference test was a quantitative PCR for M. ulcerans insertion sequence IS2404 on centralized PCR system. This has been updated in the abstract. Line 34-35

3- Introduction, lines 59-60. Indicate reference test(s) in those studies.

**Response**: The current gold standard for *M. ulcerans* detection is quantitative PCR for IS2404. These studies used this method to evaluate sensitivity and specificity of different sample types and compared the results to culture and microscopy. We believe the PCR is still the reference test in this case. We referenced these articles to justify why we used it in our study as reference test whiles noting its limitations.

4- Materials and Methods. The manuscript would benefit of adherence to STARD guidelines. A STARD workflow would be very helpful to understand the different processes.

**Response:** A STARD checklist has been attached as supplementary file and a STARD workflow included in the method section.

5- Materials and Methods, line 90. what is the author definition for a Phase II diagnostic evaluation study?

Response: Phase II was used to refer to the second stage of the assay development process (evaluation after development) to avoid confusion with a clinical trial process, ‘phase 2’ has been deleted. Line 95

6- Materials and Methods, lines 93-96, please provide the formula or a reference.

Response: Reference has been provided. Line 104

7- Note that the study was conducted with well characterized stored samples, and this is a limitation for the assessment of the real diagnostic performance.

Response: This is well noted, but for a rare disease like Buruli ulcer stored clinical samples are very important for the evaluation of new diagnostics tools especially if you require a good sample size for statistical power. This limitation has been indicated in the manuscript. Line 424 - 428

8- Materials and Methods, line 115. 200 uL sample or sample eluted/diluted in a buffer, what would be the proxy to understand the real amount of clinical sample used?

Response: Buruli ulcer samples are either swabs or fine needle aspirates. The real amount from this sampling technique is difficult to quantify – dry swabs are normally soaked in a buffer to get the cells/samples in a solution prior to extraction, similarly, FNA are rinsed in a buffer as a standard practice. 200 uL of such a suspension is a proxy of the amount of clinical samples collected from lesions.

Sentence changed to samples suspended in buffer. Line 128

9- Materials and Methods, line 125-126. Indicate elution volume

Response: Line modified to indicate the elution volume. Line 137

10- Materials and Methods. Did the authors used the same amount of DNA in the conventional real-time PCR and the strips-based real-time PCR? this is not clear and is needed for proper comparison.

Response: The same amount of DNA 2uL in a final reaction volume of 20 uL was used for both PCR methods.

11- Results, line 275. As the study samples were characterized previously by PCR, this increases pre test probability and overestimates sensitivity and my underestimate specificity. Relate this to my comment 7.

Response: Authors agree that these were samples previously characterized by PCR and the limitation duly acknowledge, but these samples were blindly tested by each laboratory with the new assay to avoid any bias. We do not believe the sensitivity was overestimated or specificity underestimated because samples were retested with both PCR methods deal with storage deterioration issues.

12- Discussion, lines 314-315. THis was done in a panel of well-characterized stored clinical samples. This has limitations, see comments 7 and 11.

Response: Comments well noted, but using well-characterized samples does not discount the diagnostic accuracy of the new assay especially when samples were retested with both the index test and the reference test.

13- Discussion, lines 339-340. As it seems developers of the method (adapted to the Biomeme system) were involved in this evaluation, I don't think it can said that evaluation was independent.

Response: Developers of the assay and the PCR platform (Biomeme) were not directly involved in testing of clinical samples. Their role was to adapt of wet-reagent PCR assay into a dried-reagent format adaptable to the portable PCR platform. Once the reagents were supplied to individual laboratory, they played no role in testing or analysis of data.

**General comment:**

We congratulate the authors on a research topic that is relevant in several ways. It is one of the research priorities for Buruli ulcer control. This study addresses several aspects of control, such as decentralizing molecular biology to improve access to rapid diagnostic confirmation for affected individuals, improving patient management and reducing the cost of care.

**Specific comments:**

**Ethical considerations**: Taking into account that this study involving human beings is multi-centre, especially multi-country, ethical approval should normally be obtained in each country. The authors only refer to the approval obtained in Ghana. What about Cameroon?

**Response**: Ethical approval information for Cameroon has been included in the manuscript. Line 107-108

The first two phases were carried out in reference laboratories. Are the authors not planning a third phase of the study? Since they believe that this innovative diagnostic tool has the potential to be fully used in primary care settings, wouldn't it make sense to test them at this level of care as well?

Response: Authors plan the carry out a prospective study of evaluating this tool at the point of patient care (primary care setting).

The limiting factor, not the least, for the expansion of this technology is the cost per test ($20-25). Innovation obviously has a cost. But from an operational point of view, who will be able to pay for this test when we know that the people affected are generally poor and live in disadvantaged environments? This is a crucial aspect that should be considered by the authors. They only mentioned that an alternative, cheaper extraction kit could be used, without saying more.

Response: Authors totally agree with reviewer that the cost is a major limiting factor. Authors are exploring other cost-effective field-friendly sample preparation method in planning the field evaluation which is the logical step as suggested by reviewer.

We look forward to meeting the high standard of PLOS Neglected Tropical Diseases.

Yours sincerely,

Dr. Michael Frimpong

(Corresponding author)
